# Intravitreal Brolucizumab Use for Retinal Vascular Diseases in Resource-Constrained Settings: A Retrospective Study From Bangladesh

**DOI:** 10.7759/cureus.103620

**Published:** 2026-02-14

**Authors:** Muhammad Moniruzzaman, Mohammed Azzam, Mst Sayedatun Nessa, Abrar Ahmed, Tangil Ahmed, Mizanur Rahman

**Affiliations:** 1 Ophthalmology, Vision Eye Hospital, Dhaka, BGD; 2 Ophthalmology, Indira Gandhi Memorial Hospital, Male, MDV; 3 Pathology, Northern International Medical College, Dhaka, BGD; 4 Ophthalmology, Kumudini Women's Medical College, MIrzapur, BGD

**Keywords:** anti-vascular endothelial growth factor, anti-vegf therapy, brolucizumab, real-world experience, retinal diseases, retinal vascular diseases, vascular

## Abstract

Background

Anti-Vascular Endothelial Growth Factor (VEGF) therapy has been proven effective and safe for retinal vascular diseases, but the use of this agent is scarce in resource-constrained countries, including Bangladesh. Considering the limited literature, we have described 17 cases and their outcome using an anti-VEGF agent (brolucizumab) for retinal vascular diseases.

Methods

This retrospective case review described patients undergoing intravitreal brolucizumab at a tertiary eye care center in Bangladesh from January 2022 to December 2023. Eighteen eyes of 17 patients with complete best corrected visual acuity (BCVA) data for four follow-ups were included. Outcomes of the treatment over six months were changes in BCVA and optical coherence tomography (OCT) measurements, including foveal center point thickness, central subfield retinal thickness, and macular volume.

Results

Ten eyes were refractory cases who switched from other anti-VEGF agents, while eight were treatment naïve. The median age of the patients was 56 years (Interquartile range or IQR: 48-68 years). Out of all, 52.9% were female patients (n=9) and 47.1% were male patients (n=8). Diabetic retinopathy (n=10, 55.6%) was the most common indication, followed by age-related macular degeneration (n=6, 33.3%). Average BCVA improved in both naïve and refractory eyes over the course of treatment. Median OCT measurements showed a decrease in both naïve and refractory cases. No adverse events occurred.

Conclusion

Brolucizumab demonstrated visual gain in the short term with a favorable safety profile in a series of cases in the real-world settings of Bangladesh. Larger studies with extended follow-up can further elucidate its long-term utility.

## Introduction

Retinal diseases characterized by aberrant vascular growth and leakages, such as diabetic retinopathy (DR), retinal vein occlusion (RVO), choroidal neovascularization, and neovascular age-related macular degeneration (ARMD) are leading causes of moderate and severe vision loss worldwide [1]. These result in macular edema, hemorrhage, exudation, and ultimately blindness when left untreated. DR and ARMD are the main causes of blindness and low vision after cataract in Bangladesh [2].

Anti-vascular endothelial growth factor (anti-VEGF) therapy has been a major advance in the management of these ocular problems [3]. VEGF antagonists inhibit vessel growth and neovascularization. Some agents can also prevent the formation of pathological microvessels, stabilize normal vasculature, and suppress inflammation [3]. Intravitreal injections (IVI) of anti-VEGF agents like ranibizumab, bevacizumab, and aflibercept are now commonly used first-line therapies to target VEGF-driven vasculopathy and improve outcomes [4-7].

Although the commonly used anti-VEGF therapy has been shown to provide highly effective therapy, these often require frequent injections [4]. Additionally, a proportion of patients develop refractory diseases unamenable to these traditional agents [8]. This refractory disease poses a challenge to the optimization of treatment regimens. Switching anti-VEGF regimen has been used to improve response in some cases [8]. Brolucizumab is a novel anti-VEGF agent approved for intraocular use by the United States Food and Drug Administration (FDA) and the European Medicines Agency (EMA) [9]. The approval was based primarily on the results of two large phase three, multicenter, randomized HAWK and HARRIER clinical trials [10]. With its small molecule size, high affinity, and potency, it has shown superior resolution of fluid on optical coherence tomography compared to aflibercept in these trials. It also allows for a lower dosing schedule in the majority of patients, providing an advantage over other anti-VEGF agents [11,12]. Moreover, adverse events like intraocular inflammation were recorded in relatively fewer cases compared to other agents [10,13].

Real-world data on the utilization of brolucizumab as a switch therapy in anti-VEGF refractory cases or as a first-line agent can provide useful insights because, compared to clinical trials, patients remain subject to multifarious unmeasured factors that could influence the outcome of the treatment in day-to-day clinical practice. Particularly, the experience of users from different ethnic backgrounds could capture the variations in the treatment effectiveness [14] across regions. The studies reporting the experience of using intravitreal brolucizumab to date have mostly been published from high-income countries, with very few from low- and middle-income countries like India [11,15]. These studies have shown overall improvement in visual acuity and retinal anatomy. In addition, a distinct risk of intraocular inflammation among the treatment recipients has been reported, raising safety concerns [15].

In the context of Bangladesh, where nearly 73% of health expenditure is spent out-of-pocket, the treatment might not be affordable for everyone because of the cost of brolucizumab [16]. Moreover, the scarcity of vitreo-retinal experts with their concentrated presence in the capital makes the treatment inaccessible for many. Therefore, reporting the real-world effectiveness of the intravitreal brolucizumab injection could enrich the knowledge base and inform clinicians on its usage among people of Bengali ethnicity. The purpose of this study was to evaluate the short-term visual and anatomical outcomes and safety profiles of intravitreal brolucizumab from a series of retinal disease cases treated with the agent over six months in a real-life clinical setting in Bangladesh.

## Materials and methods

Study population, site, and duration

**Figure 1:**
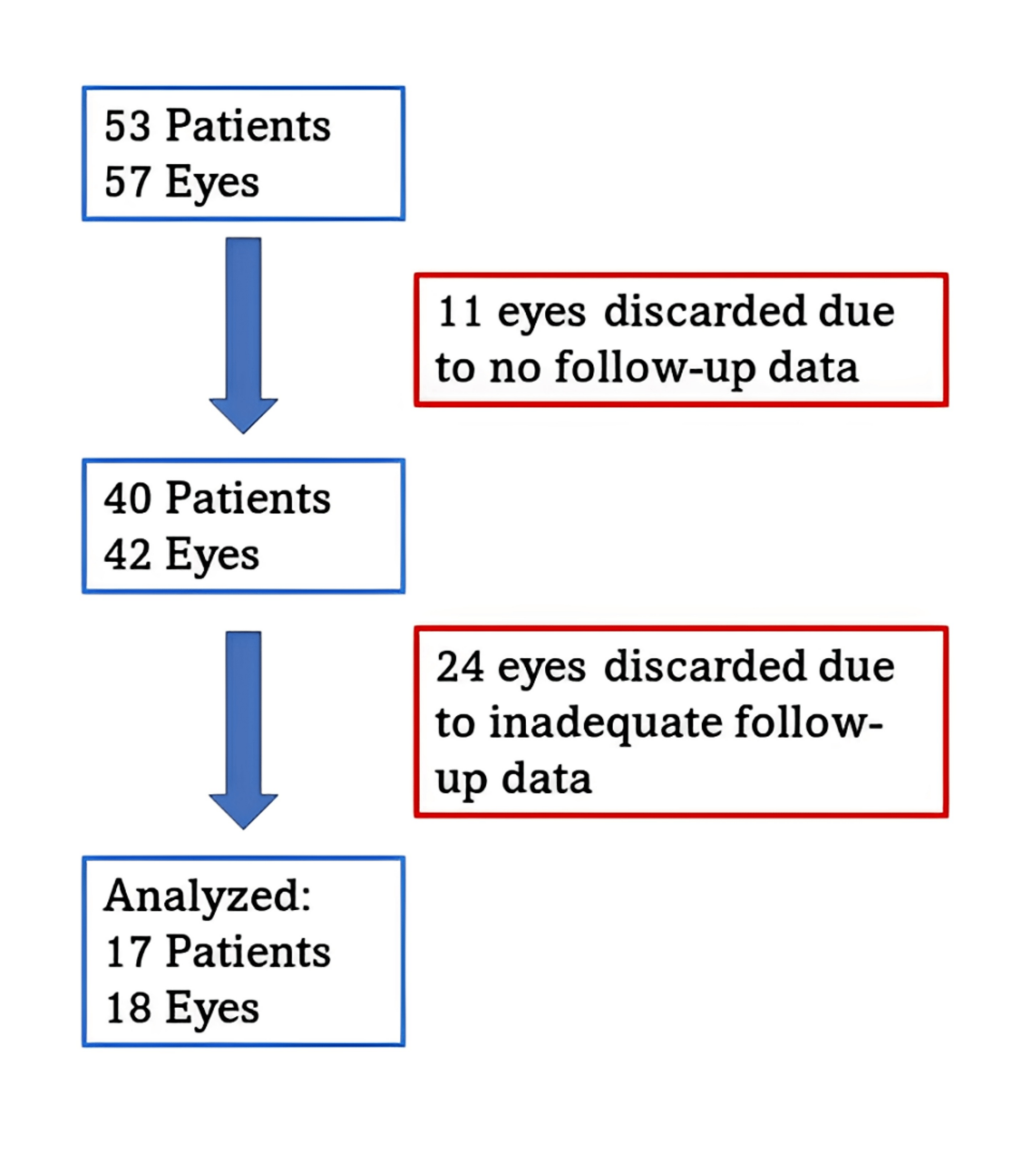
Flowchart of patient and eye selection

This study reviewed the records of patients who underwent IVI brolucizumab at a tertiary eye care center (Vision Eye Hospital) in Bangladesh between January 2022 and July 2023. Data was extracted from a Microsoft Excel file (Microsoft Corp., Redmond, WA, USA), where patient information was being recorded. The extraction was done by the principal investigator and checked by the co-investigators. The records of a total of 58 eyes from 53 patients who had received IVI brolucizumab for macular edema were screened for inclusion. Finally, only 19 eyes of 17 patients who had complete best corrected visual acuity (BCVA) data for four follow-ups were included. Eleven eyes were excluded due to a lack of one or more follow-ups, and 14 eyes were excluded due to incomplete data (Figure 1).

Incidentally, we found eight treatment-naïve and 10 refractory eyes treated with brolucizumab.

Sociodemographic information and measurement of retinal anatomy

A semi-structured form was used for data collection. Baseline data collected included age, sex, diagnosis with duration, indications for anti-VEGF treatment, history of prior anti-VEGF injections, BCVA measured on a Snellen chart, and foveal center point thickness (FCP), central subfield retinal thickness (CSRT), and macular volume (MV) were recorded from the optical coherence tomography (OCT) measurements. FCP and CSRT represent retinal thickness measurements at the fovea and the central 1 mm circle, respectively [17]. The macular volume comprises the sum of all zone volumes within the central 3 mm and 6 mm circles [18].

Outcome measurements

The total dose of brolucizumab injections to be given to each eye was decided on clinical judgement. The loading doses (three doses per eye) were provided at one-month intervals for patients with ARMD, and at six-week intervals for DR and RVO. The follow-up durations were determined based on guidelines [19] and clinical judgement. For patients who did not respond at follow-ups, additional doses were provided. The main outcomes taken from the records were changes in BCVA and OCT measurements at follow-ups. Follow-ups were done at 30-day intervals for patients with clinically significant macular edema (CSME) and diabetes and 45 days for other abnormalities. A total of four visits were considered. BCVA data were available for all four follow-ups in the 19 eyes included. However, OCT measurements were available only for nine eyes at the third and fourth follow-ups. We also checked the records for any adverse events.

Data analysis and presentations

The baseline and follow-up information of the participants were presented as summary statistics, where categorical variables were expressed as frequency (percentage) and continuous variables were expressed as median (Interquartile Range or IQR) and mean (SD). All eye measurements (n=19) were analyzed independently, irrespective of bilateral involvement in a person. BCVA was converted to a logarithm of the minimum angle of resolution (logMAR) using the Excel sheet published and made available by Moussa et al. [20] for easy interpretation and readability. Differences between BCVA and OCT measurements at baseline between treatment-naïve and refractory groups were assessed using the Mann-Whitney U test. A complete case analysis approach was taken for the evaluation of changes in visual acuity and retinal anatomical measurements by excluding cases with missing data in any of the follow-ups. Changes in BCVA and OCT measurements were tested through a mixed-method repeated measures analysis of variance and were presented through line plots. A p-value of <0.05 was considered significant for all statistical tests. Statistical analysis was carried out in the statistical software IBM SPSS Statistics for Windows, Version 26 (Released 2019; IBM Corp., Armonk, New York, United States).

Ethical considerations

The study protocol was reviewed and approved by the Institutional Review Board (IRB) of the Vision Eye Hospital, Bangladesh (VEH/IRB/2021/11). The authors declare that no human subjects were harmed and the procedures followed were in accordance with the ethical standards and regulations established by the Helsinki Declaration. As patient consent was taken for the usage of the data for research at the time of treatment initiation, additional consent was not sought.

## Results

Patient characteristics and pre-brolucizumab features

The median age of the participants was 56 years (IQR: 48 - 68), with nine female patients (52.9%) and eight male patients (47.1%). Out of 19 eyes explored, 12 (63.2%) were right eyes, and seven (36.8%) were left eyes. All the patients had dimness of vision, nine had CSME, and one patient had vitreous hemorrhage. DR was the most common indication (n=10, 55.6%), followed by ARMD (n=6, 33.3%), and RVO (n=2, 11.1%). Among all participants, 11 had diabetes mellitus (61.1%), and 10 (55.6%) had arterial hypertension. Twelve eyes were refractory (switch cases) (63.2%), and seven (36.8%) were treatment-naïve cases. Bevacizumab (35.3%) was the most administered anti-VEGF therapy. The median dose of anti-VEGF injections received a priori was 3.5 (IQR: 2.75 - 4.25). The median brolucizumab dose received was three (IQR: 3 - 5) (Table 1).

**Table 1:** Patient characteristics and pre-brolucizumab features *Multiple response; Data of 18 eyes from 17 participants, presented as frequency (percentage) and median (IQR) where appropriate;  BCVA: Best corrected visual acuity; FCP: Foveal center point: CSRT: Central Subfield Retinal Thickness; MV: Macular volume; VEGF: Vascular endothelial growth factor.

Variable	Statistics
Age (years)	56 (48-68)
Sex	
Male	8 (47.1)
Female	9 (52.9)
Eyes	
Right	12 (63.2)
Left	7 (36.8)
Clinical presentation	
Dimness of vision	18 (100.0)
Clinically significant macular edema (CSME)	9 (50.0)
Ventricular hemorrhage	1 (5.6)
BCVA (logMAR)	0.477 (0.301-0.778)
Optical Coherence Tomography (OCT)	
FCP (µm)	340.4 (305.6-356.1)
CSRT (µm)	377.0 (265.8-446.5)
MV (mm^3^)	9.6 (8.6-10.1)
Duration of illness (days)	45 (30-180)
Indications for treatment*	
Diabetic retinopathy (DR)	10 (55.6)
Proliferative diabetic retinopathy (PDR)	4 (22.2)
Non proliferative diabetic retinopathy (NPDR)	6 (33.3)
Age-related macular degeneration (AMD)	6 (33.3)
Retinal vein occlusion (RVO)	2 (11.1)
Comorbidities	
Diabetes mellitus	11 (61.1)
Hypertension	10 (55.6)
Case type	
Refractory (switch)	12 (63.2)
Treatment-naïve	7 (36.8)
Previous anti-VEGF therapy	
Any	10 (58.8)
Bevacizumab	6 (35.3)
Ranibizumab	3 (17.6)
Aflibercept	2 (11.8)
Dose	3.5 (2.7-4.3)
Brolucizumab dose	3 (3-5)

Characteristic differences between treatment-naïve and refractory cases at baseline

The median BCVA measured in terms of logMAR was 0.477 (IQR: 0.310-0.778) for treatment-naïve cases and 0.477 (0.176-0.834) for refractory cases. For treatment-naïve cases, the median FCP, CSRT, and MV were 319.2 µm (276.4-405.2), 367.0 µm (217.0-417.0), 9.0 mm^3^ (7.8-11.5), respectively. For refractory cases, these measurements were 340.6 (332.2-356.7), 434.0 (271.0-452.0), and 9.6 (9.4-10.1), respectively. Therefore, the median FCP, CSRT, and MV were relatively higher among refractory cases. However, the difference was not statistically significant (p>0.05) (Table 2).

**Table 2:** Characteristic differences between treatment-naïve and refractory casesa at baseline *p-values were determined by Mann-Whitney U test; Data of 18 eyes from 17 participants, presented as n(%) and median (IQR) where appropriate. AMD: Age related macular degeneration; BCVA: Best corrected visual acuity; FCP: Foveal center point: CSRT: Central Subfield Retinal Thickness; MV: Macular volume; NPDR: Non-Proliferative Diabetic Retinopathy; OCT: Optical Coherence Tomography: PDR: Proliferative Diabetic Retinopathy; RVO: Retinal Vein Occlusion.

Variable	Treatment-naïve (n=7)	Refractory (n=12)	p-value*
Visual acuity (n=18)			
BCVA (logMAR)	0.477 (0.301 – 0.778 )	0.477 (0.176 – 0.834)	1.000
OCT measures (n=8)	(n=5)	(n=3)	
FCP (µm)	319.2 (276.4 – 405.2)	340.6 (332.2 – 356.7)	0.583
CSRT (µm)	367.0 (217.0 – 417.0)	434.0 (271.0 – 452.0)	0.510
MV (mm^3^)	9.0 (7.8 – 11.5)	9.6 (9.4 – 10.1)	0.583

Outcome of treatment with brolucizumab

**Figure 2:**
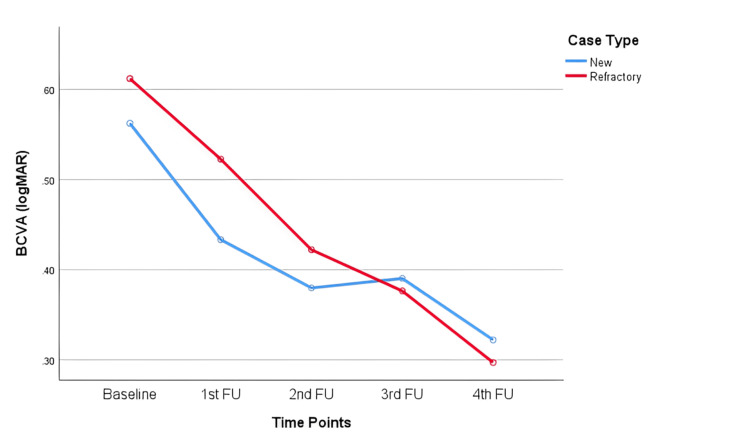
Changes in best corrected visual acuity measured in logMAR from baseline to six months after the treatment in new and refractory cases Mixed ANOVA revealed a significant improvement in visual acuity (within-subject effects; p<0.001) over time in both new and refractory cases. However, the difference in improvement between these two groups (between subject effects) was not significant (p=0.802).

The average BCVA improved from the pre-brolucizumab stage (baseline) to six months (fourth follow-up) after initiation of brolucizumab treatment in both new and refractory cases (p<0.001) (Figure 2 and Table 3).

**Table 3:** Changes in visual acuity and optical coherence tomography (OCT) measurements from baseline and follow-up BCVA: Best corrected visual acuity; FCP: Foveal center point; CSRT: Central Subfield Retinal Thickness; MV: Macular volume.

Assessment	Case type (n)	Timepoints mean (SD)				
		Baseline	FU1	FU2	FU3	FU4
BCVA (logMAR)	Treatment- naïve (n=7)	0.793 (0.5)	0.752 (0.891)	0.451 (0.476)	0.492 (0.5)	0.392 (0.534)
	Refractory (n=12)	0.789 (0.672)	0.656 (0.593)	0.505 (0.659)	0.506 (0.432)	0.427 (0.528)
FCP (µm)	Treatment-naïve (n=3)	376.5 (71.12)	-	-	289.1 (18.32)	278.83 (28.38)
	Refractory (n=6)	345.2 (27.95)	-	-	326.52 (41.34)	318.17 (85.47)
CSRT (µm)	Treatment-naïve (n=3)	328 (142.56)	-	-	335.33 (106.08)	244 (96.39)
	Refractory (n=6)	382.67 (182.01)	-	-	366.67 (175.07)	312.33 (179.28)
MV (mm^3^)	Treatment-naïve (n=3)	10.64 (2.02)	-	-	8.18 (0.52)	7.89 (0.8)
	Refractory (n=6)	9.76 (0.79)	-	-	9.23 (1.17)	8.96 (2.43)

**Figure 3:**
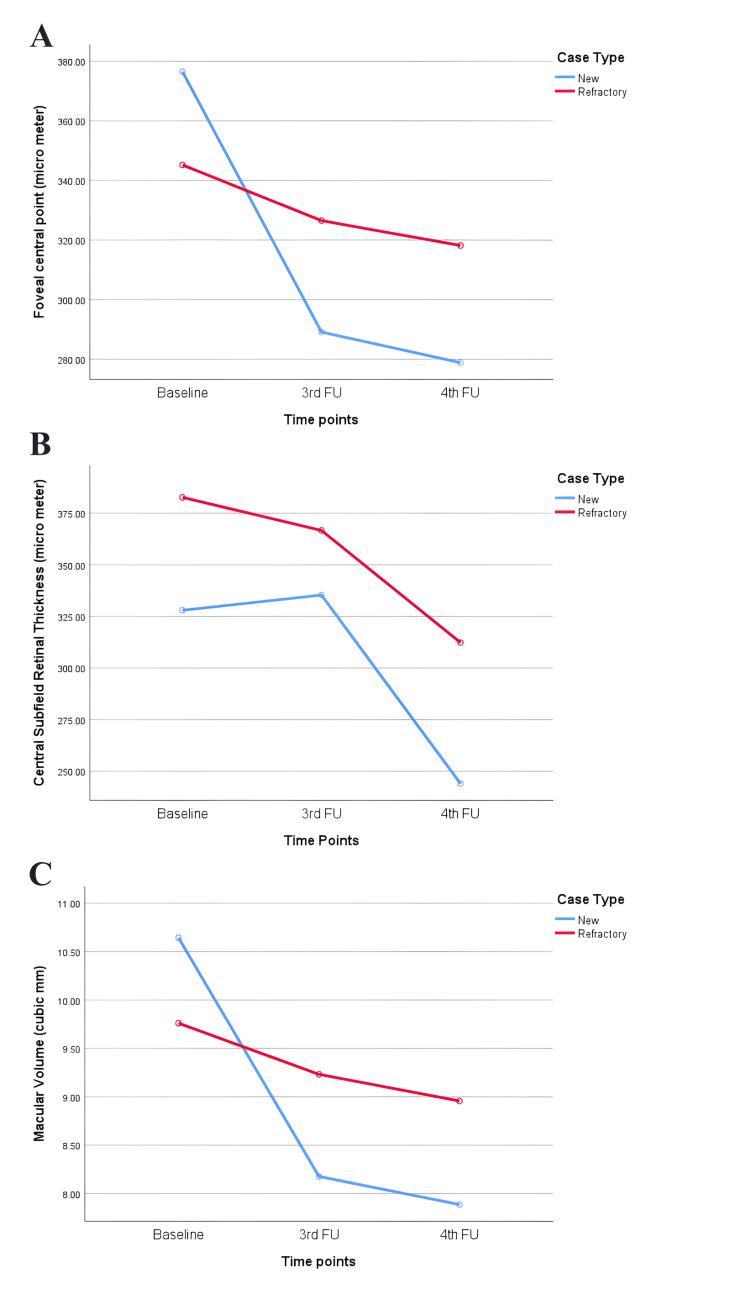
Changes in (A) focal central point, (B) central subfield retinal thickness, and (C) macular volume from baseline to third and fourth follow-ups after treatment with brolucizumab Mixed ANOVA revealed improvement in optical coherence tomography (OCT) measurements (within-subject effects; (A) p=0.061, (B) p=0.645, (C) p=0.060) over time in both new and refractory cases. The difference in improvement between these two groups (between subject effects) was not significant ((A) p=0.551, (B) 0.317; (C) p=0.565).

The OCT measurements for baseline, third, and fourth follow-ups were available for nine cases only. Among them, three were new, and six were refractory cases. The median FCP and MV (measured at OCT) decreased in all these cases at three to 4.5 months (third follow-up) and four to six months (fourth follow-up) after the brolucizumab injection (Figure 3 and Table 3).

On average, CSRT showed a consistent decrease in refractory cases, with a slight increase at the third follow-up and then a decrease at the fourth follow-up in new cases.

**Figure 4:**
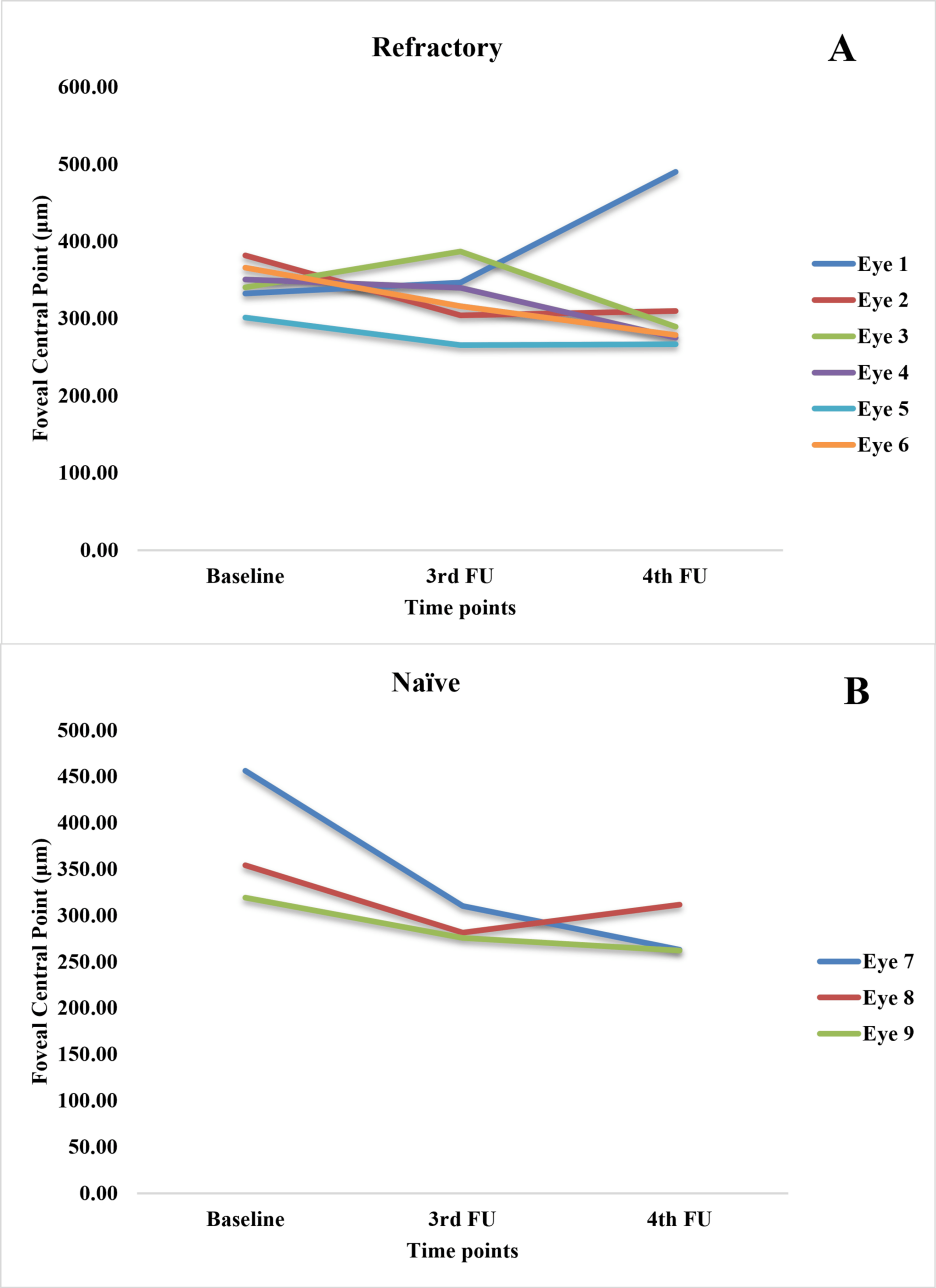
Changes in the foveal central point thickness in (A) refractory and (B) naïve cases from baseline to the third and fourth follow-ups

**Figure 5:**
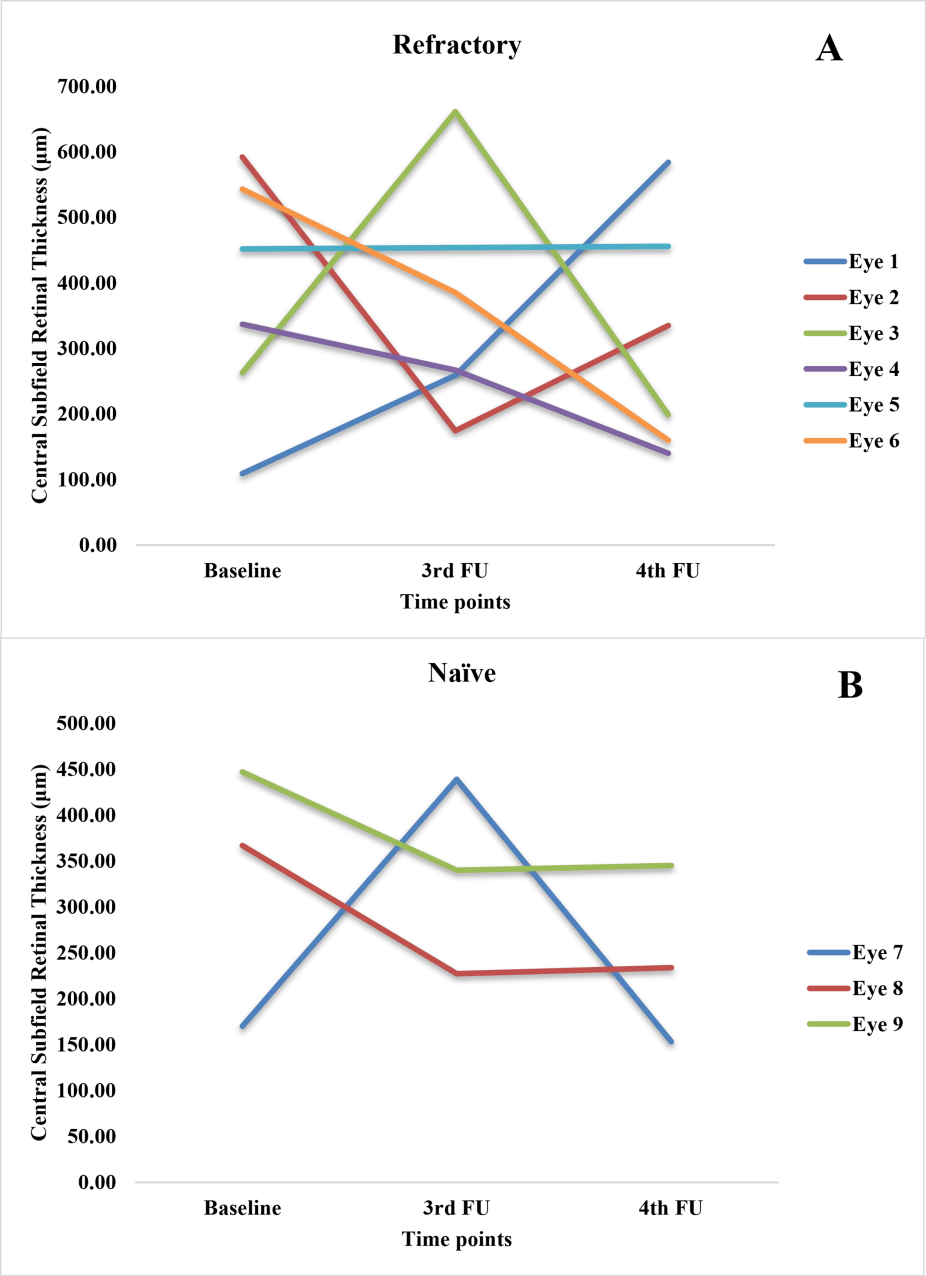
Changes in the central subfield retinal thickness in (A) refractory and (B) naïve cases from baseline to third and fourth follow-ups

**Figure 6:**
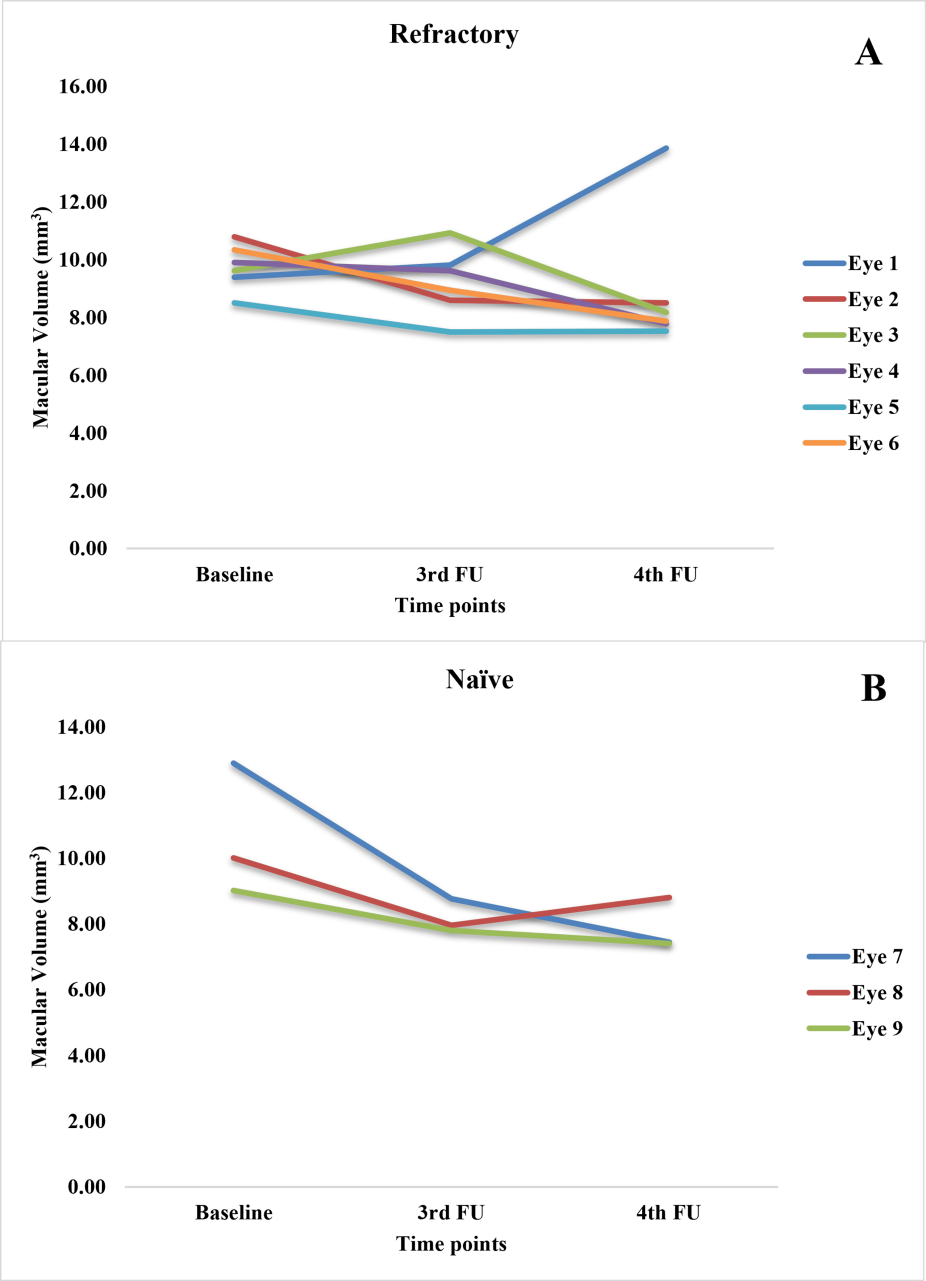
Changes in the macular volume in (A) refractory and (B) naïve cases from baseline to the third and fourth follow-ups

Figures 4-6 show the case-wise changes in FCP, CSRT and MV among the nine eyes.

In FCP measurements, three refractory eyes (two, five, and six) showed a consistent decrease over three follow-ups. Among the rest of the refractory eyes, three and four showed an increase at third follow-up with a decrease at the final follow-up. Only eye one showed a continuous increase in FCP, pointing to a non-response. Regarding CSRT, the values decreased consistently in eyes four and six, showed an increase followed by a decrease in eye three, a persistent increase in eyes one and five, and a decrease followed by an increase in eye two. Among refractory cases, eyes seven and nine showed a persistent decrease, while eye eight showed an increase followed by a decrease. In MV measurements, eyes two and five showed a decrease followed by stasis; eye six showed a consistent decrease; eyes three and four showed an increase followed by a decrease, and eye one showed a persistent increase. Among naïve cases, eyes seven and nine showed a consistent decrease, while eye eight showed a decrease followed by a slight increase.

Adverse events

No adverse events, including ocular inflammation, were recorded in any of these 18 eyes within six months after treatment with brolucizumab.

## Discussion

Brolucizumab has been demonstrated to be an effective and safe agent against VEGF-driven neovascularisation-associated diseases like ARMD [21], DR, and macular edema [11], and central serous chorioretinopathy [22]. This study reports the short-term (six months) effectiveness and safety of brolucizumab among a series of 19 eyes with retinal vascular diseases in a real-world setting. Notably, many patients were lost to follow-up, possibly due to cost and the distance of the center from patients’ residences. The setting where the injections were provided is a private hospital, and all treatment costs were borne by the patients. Given the price of each dose of brolucizumab in the country (~260$), it is difficult for every patient to carry on with the treatment. Also, the center is located in the capital city of Dhaka, where patients come for treatment from all over Bangladesh. Hence, accessibility to the healthcare facility could have been a barrier to continuing follow-up and treatment.

We noted that refractory cases had relatively higher median OCT values, probably indicating a higher severity of the underlying pathology [23-25]. A higher FCV, CSRT, and MV indicate central thickening, macular edema, and diffuse thickening or cysts, respectively. 

We treated both treatment-naïve and refractory eyes with brolucizumab. The median dosage required was three, and it was given as a switch therapy in refractory eyes. Overall, six cases required additional doses after the three loading doses. Among them, two were refractory. Refractory cases had previously been treated with bevacizumab, ranibizumab, and aflibercept with a median dose of 3.5. In our study, eyes showed significant improvement in BCVA over four to six months in both new and refractory cases. Although new cases found a significant improvement in retinal thickness measurements, implying a reduction in retinal fluids and anatomic corrections over six months, some refractory cases experienced a relapse in fluid accumulation after 4.5 months. The CSRT measurements also revealed that macular edema was completely resolved in treatment-naïve cases (<300 micrometers), but a small amount remained in refractory cases. The non-response and relapse in refractory cases could be attributed to the initial severity of the underlying pathology and control of comorbid conditions (e.g., diabetes) during treatment, or often due to acquired resistance to anti-VEGF therapies [26]. Although we found a higher degree of macular edema among refractory cases, none of the other reasons could be evaluated in the context of this study.

The findings of our study reflect the OCT findings of other studies in that the majority of the previous real-world studies on treatment-naïve and switch eyes with ARMD showed significant improvement in BCVA and central subretinal thickness measurements at three months to 12 months after initiation of brolucizumab [12,21,27,28]. Additionally, compared to phase 3 trials, real-world studies found promising results with fewer intraocular inflammation events with this agent [21]. Similarly, brolucizumab showed significant improvement in BCVA and CSMT in patients with DR in a large number of real-world studies and clinical trials [11]. We treated 10 cases of DR, six cases of ARMD, and two cases of RVO during the study period. The RVO were refractory cases, whereas nearly half of the DR and ARMD cases were treatment naïve. 

Most of the real-world studies conducted on ARMD reported outcomes at three months after initiation of treatment. Whereas our study reports a finding over a longer period and shows a sustained response in visual acuity in both treatment-naïve and refractory cases. The OCT measurements also showed a similar improvement over 4.5 months in all cases, irrespective of the refractoriness of the treated eyes. However, refractory cases experienced a median increase in CSRT and MV at the third follow-up, probably because of the increase in one non-responding case where the CSRT continued to increase and another case in which the CSRT increased at the third follow-up but decreased at the final follow-up. Non-responders may often show immediate deterioration in visual outcomes after switching to brolucizumab therapy, as noted by Yeom et al. [29]. However, we saw such deterioration in only one case. 

Our patients were given brolucizumab over four- to six-week intervals (30 to 45 days) based on indication and clinical judgements. Saba and Walter found a median prolongation of intervals after switching to brolucizumab [30]. However, a longer interval in some cases could have led to an increase in OCT measurements in our study. Chakraborty et al. [28] have shown that early recurrence in fluid may occur as early as 16 weeks after a single dose of brolucizumab. However, as all our patients received three loading doses, the ones showing no or slow improvements were provided additional doses; the effect of the interval variations could not be evaluated.

Among all patients, only one refractory eye failed to respond to brolucizumab switch therapy. The patient was a nondiabetic and non-hypertensive 80-year-old male subject who had developed dimness of vision due to choroidal neovascular membrane associated with ARMD. He had received three doses of ranibizumab before switching to brolucizumab. The failure of ranibizumab to produce an adequate response in this individual endorses the importance of age as a factor behind treatment failure [31].

None of our studied eyes observed any intraocular inflammation events within six months, which is in contrast to all real-world observational studies [15,21] where these events were found to be common. As adverse events were not recorded in a systematic manner, and many patients were lost to follow-up, we could have missed the adverse events.

The limitation of this real-world case series was that only 19 eyes could be studied. The OCT measurements were available over the follow-up period for only nine eyes, making any conclusive remark on the management of refractory cases based on brolucizumab difficult. Also, as the mixed-method ANOVA was applied to a small sample size, the results (particularly, the between-subject heterogeneity) should be interpreted with caution. Among the patients, six required additional doses in addition to the loading dose. Among the nine patients for whom OCT measurements were shown, all the three treatment-naïve cases received one additional dose, and one of the six refractory cases received two additional doses. Hence, the changes should be read in the context of patients, rather than the loading dose. The effect of comorbidities on the treatment outcome could not be evaluated because of a lack of records. The qualitative assessment of retinal fluid accumulation by vitreo-retinal experts was not available from the records. The effect of systemic comorbidities on the effectiveness of the drug could not be evaluated due to lack of data. A major limitation of the study is that adverse events were recorded on the clinician’s judgement, and no standardized definition or checklist was followed. Hence, these could have been underreported. Moreover, the possibility that patients with intraocular inflammation were missed due to follow-up loss cannot be discounted. However, this was one of the earliest studies from a low-resource setting and among Bengali ethnicity that could provide insight into the real-world effectiveness and safety of the newer anti-VEGF agent brolucizumab in this population.

## Conclusions

In this retrospective study, intravitreal brolucizumab demonstrated significant short-term improvement in visual acuity over four to six months in both treatment-naïve and anti-VEGF refractory cases of retinal vascular diseases. However, refractory cases showed either a resistance to fluid reduction or a recurrence of fluid after initial drying, which could have diminished the effect of overall improvements. No adverse events were found in the record during the study period. However, because of the small sample of eyes with incomplete data, the findings should be read with caution. Nevertheless, the real-world data that this study provided could be used to understand the response patterns in routine clinical practice. Future studies should consider a large sample, multi-center, prospective, post-marketing surveillance studies with systematic recording of adverse events for proper evaluation of the effectiveness and safety of the brolucizumab in specific indications. Longer follow-ups are warranted to elucidate long-term visual and anatomic outcomes, optimal dosing, and safety profile of brolucizumab in routine clinical practice.
